# Role of ultrasound in detection of radiolucent foreign bodies in extremities

**DOI:** 10.1007/s11751-018-0308-z

**Published:** 2018-02-09

**Authors:** Mehraj D. Tantray, Asim Rather, Qazi Manaan, Irfan Andleeb, Mir. Mohammad, Yasmeena Gull

**Affiliations:** 1 Department of Orthopaedics, Bone and Joint Hospital, Barzulla, GMC, Srinagar, Jammu and Kashmir 190005 India; 2 Department of Radiognosis, GMC, Srinagar, Jammu and Kashmir India; 3 Department of Health and Family Welfare J&K Health Services India, Srinagar, India

**Keywords:** Extremities, Foreign body, Radiolucent, Ultrasonography

## Abstract

Removal of foreign bodies from soft tissues in emergency is very challenging and becomes more problematic when it is radiolucent. Blind exploration is sometimes hazardous for patients especially when it is in proximity to a vessel or a nerve or an overlying tendon. The purpose of this study was to determine the accuracy of ultrasonography (USG) in detecting radiolucent soft tissue foreign bodies in the extremities. From January 2014 to January 2016, 120 patients with either a positive history or clinically suspected soft tissue foreign body and negative radiography were evaluated by USG with a high-frequency (13–6 MHz) linear-array transducer. The sonographic findings were used to guide surgical exploration. Out of 120 patients who underwent surgical exploration, USG was positive in 114 cases, and foreign body was retrieved in 108 cases, and among the six cases where USG was negative, foreign body was retrieved from one case. In one case with strong clinical suspicion of foreign body USG was falsely negative. Majority of foreign bodies were removed from foot (69 cases) and hands (26 cases), and rest of foreign bodies were removed from ankle (4 cases), wrist (3 cases), thigh (2 cases), leg (1 case), knee (2 cases), forearm (2 cases). Accuracy, sensitivity, and positive predictive value were determined as 94.16, 99.08, and 94.13%, respectively. The real-time high-frequency USG is a highly sensitive and accurate tool for detecting and removing radiolucent foreign bodies which cannot be visualized by routine radiography.

## Background

Penetrating foreign bodies are common in patients visiting emergency departments [1, 2]. Farming being the most common job among the population in Kashmir with practice of working bare footed in orchards, the probability of penetration of tree splinters as a radiolucent foreign body in the extremities, especially the sole of the foot, is high. The missed foreign body may remain asymptomatic for prolonged periods or else lead to a wide range of complications including pain, abscess, chronic discharging wound, necrotizing fasciitis bone and joint destructive lesions granulomas with impairment of tendon mobility or triggering of digits migration delayed tendon ruptures, neurodeficits, pyogenic granulomas, vascular events, massive soft tissue injury, and lawsuits [3–15].

A radiolucent foreign body such as wood frequently remains undetected [16]. Sonography plays an important role in the evaluation of these patients [17].

Sonography has a reported sensitivity of 95% for detection of foreign bodies [18, 19].

In previous reports the positive predictive value of conventional radiography (CR) and sonography (US) were 100 and 95%, respectively, and for computed tomography (CT), and magnetic resonance imaging (MRI) were 95 and 93.8%, respectively. CT had a negative predictive value of 78.3%, while US, MRI, and CR had 73.7, 70.1, and 53.7%, respectively [20].

Non-opaque foreign bodies are visualized as hyper-echoic foci with accompanying acoustic shadows [17]. A hypoechoic halo surrounding the foreign body is sometimes seen, which represents edema, abscess, or granulation tissue [21].

The purpose of the study was to determine effectiveness of sonography for detection of radiolucent foreign bodies and to summarize the experiences using sonography in the management of patients with a suspected retained foreign body.

## Materials and methods

**Fig. 1:**
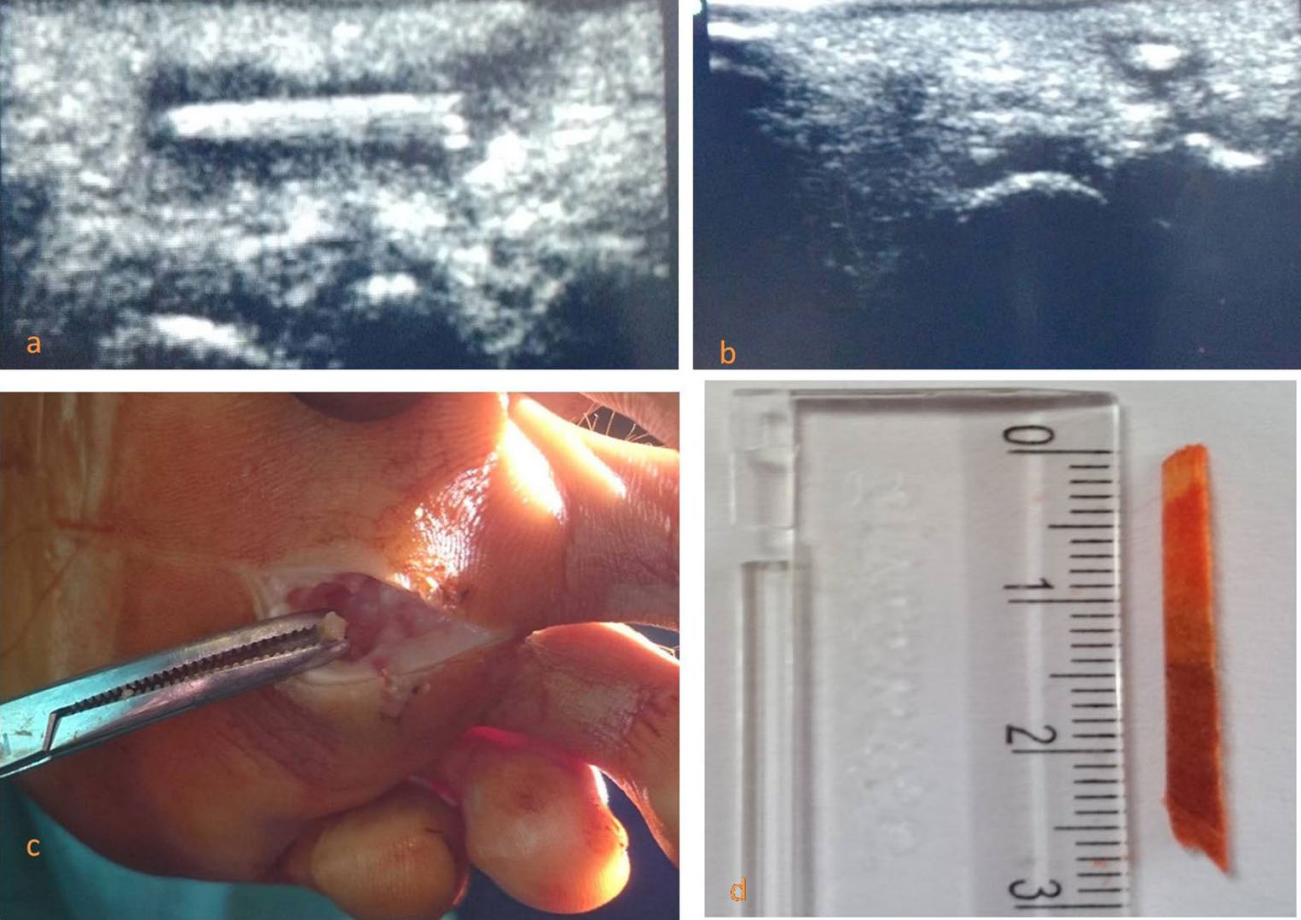
Ultrasound of foot of the patient with chronic discharging sinus (**a** & **b**). Surgical removal (**c**) and wood foreign body (**d**)

From January 2014 to January 2016, 120 symptomatic patients with definite history and clinical suspicion of soft tissue foreign body and negative radiography were included in the study. A single radiologist who had 6 years of experience in the radiology department received the radiographs and carried out the sonographic examination. All ultrasound examinations were carried out on a sonosite Micromaxx USG machine using high-frequency (13–6 MHz) linear-array transducer. USG scans were performed in multiple planes as required according to the part being examined. After location of the soft tissue foreign body, its size, depth, and orientation were documented. Relationship to other structures such as muscles, tendon, bone, and vessels were determined. Doppler mode was also employed wherever deemed necessary. Any associated abscess, granuloma, or cellulitis were evaluated. A single orthopedic surgeon carried out direct surgical exploration guided by sonographic findings. Sensitivity of USG was determined with respect to the findings on surgical exploration (Fig. 1).

## Results

**Fig. 2:**
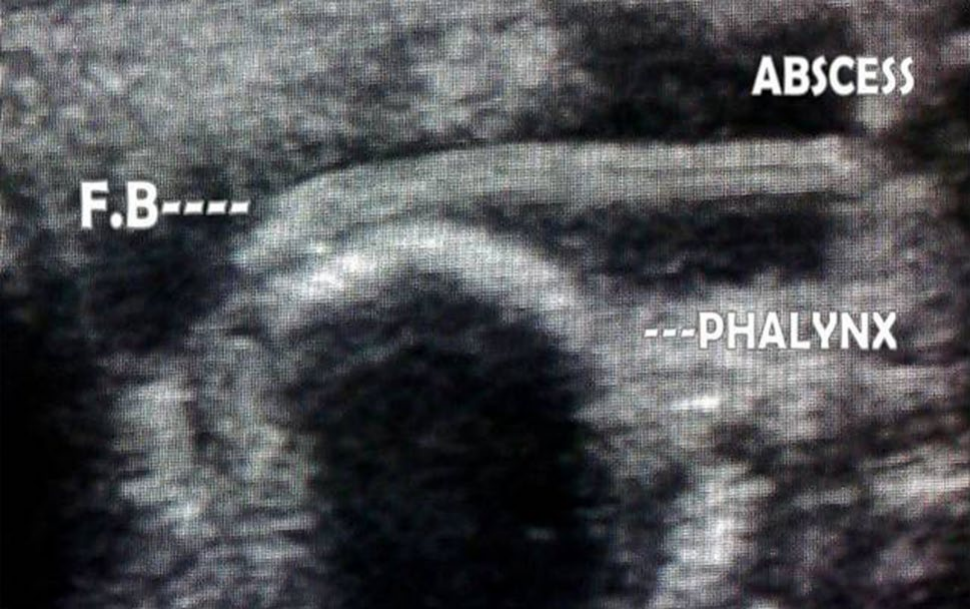
Ultrasound of hand showing foreign body with abscess formation on dorsum of hand

One hundred and twenty patients underwent surgical exploration; among them 114 patients had a positive USG, and foreign body was retrieved from 108 patients, and in the rest of six patients in whom USG was positive, five had underwent at least one previous exploration. Among the six patients in whom USG was negative, one patient with strong suspicion of foreign body had chronic discharging sinus near tendo Achilles insertion, and a thorn was removed on exploration. Ninety patients (75%) were males and 30 (25%) were females. Mean age of the patients was 27.6 years (range 6–70 years). Duration of the patients’ complaint was from 1 day to 4 years, while in 50% of cases, it was less than a month. Predominant chief complaints of the patients were: foreign body sensation in 49 (40.83%), abscess formation in 38 (31.66%), discharging wound in 19 (15.83%), and pain in 14 (11.66%) cases. Foreign bodies removed were wooden (41 cases), thorn (38 cases), rubber/plastic from nail slipper (22 cases), and glass (8 cases). Majority of foreign bodies were removed from foot (69 cases) and hands (26 cases), and rest of foreign bodies were removed from ankle region (4 cases), wrist (3 cases), thigh (2 cases), leg (1 case), knee (2 cases), forearm (2 cases) (Tables 1, 2). Size of the foreign body varied between 3 to 32 mm with mean of 15 mm (Fig. 2).

**Table 1:** Site of foreign body removal in order of frequency

Site of foreign body removal	No. of cases
Foot	69
Hands	26
Ankle	4
Wrist	3
Thigh	2
Leg	1
Knee	2
Forearm	2
Total	109

**Table 2:** Types of foreign bodies recovered

Nature of foreign body	Number of cases
Wooden	41
Thorn	38
Rubber/plastic/nail slipper	22
Glass	08
Total	109

**Fig. 3:**
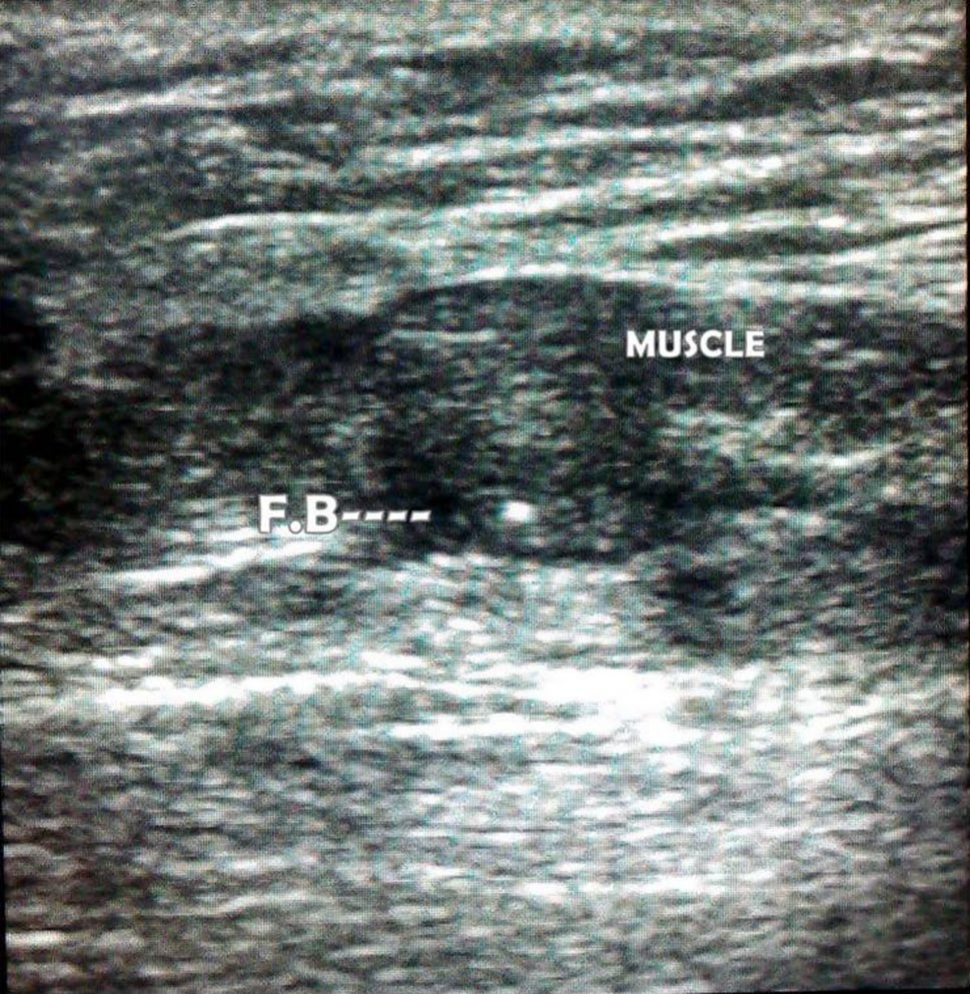
Ultrasound of calf showing foreign body within the muscle

The accuracy, sensitivity, and PPV of this study were 94.16, 99.08, and 94.13%, respectively (Fig. 3).

## Discussion

Conventional radiographs should be obtained to rule out the presence of radio-opaque foreign objects. Plain radiographs will depict approximately 80% of all foreign bodies, but several types of radiolucent foreign bodies such as wood remain undetected [22]. Plain radiographs of wooden FB are negative in 86% of such patients [23]. In these patients, sonography is the modality of choice for identification of such radiolucent foreign body.

The identification of wooden foreign bodies may be difficult on MRI, especially when foreign bodies are small, and there is no associated abscess, granulation tissue, or fluid collection. In such cases, the foreign body may appear as a signal void with surrounding nonspecific granulation tissue. Wooden foreign bodies may be seen signal void in all sequences, but after water absorption, it could be seen hypo-intense on T1 and hyper-intense on T2 images [17]. When compared with Sonography, MRI is more expensive, less readily available, and has less value in the detection of small wooden foreign bodies.

Sonographic evaluation provides important information on the depth, size, and anatomical relationship with surrounding structure [18, 21, 24]. Although CT has sensitivity 5–15 times greater than that of plain X-ray, it is not as sensitive as US, or MRI [25]. Additionally, the expense, use of radiation, and availability make the use of CT less than optimal in the clinical setting.

Out of 120 patients, USG was positive in 114 patients, and foreign body was retrieved from 108 patients, and in six patients for whom USG was falsely positive, five had underwent at least one previous surgical exploration, whereas among the six patients in whom USG was negative, foreign body was retrieved in one patient who had chronic discharge near ankle region. Detection of foreign body is difficult in interphalangeal space and in air-contaminated tissue after a penetrating trauma. FB must be distinguished from hyper-echoic body tissue such as ossified cartilage sesamoid bones, scar tissue, gas bubble, and intermuscular fascia. Acoustic shadowing is an important clue in the differential diagnosis [18, 21]. Acoustic shadowing can differentiate foreign body from scar tissue, gas bubble, and normal intermuscular fascia, because they are void of acoustic shadowing.

Our results demonstrate the effectiveness of sonography for detection of radiolucent FB. It is therefore an important and easily available modality that facilitates removal of the object by reducing the blind explorations and chances of iatrogenic tissue damage.

Peterson et al.  [17] showed that sonography is the modality of choice in patients who present with a history of antecedent skin puncture or when a penetrating injury is suspected.

Dumarey et al.  [26] showed that CT gave a good anatomic overview, but was not able to show the smaller fragments. Performing sonography is mandatory in patients with penetrating injuries by foreign bodies because it is very sensitive.

We believe that all foreign bodies were seen during sonographic examination as echogenic objects, and most of them (wooden, glass, etc.) may also show similar sonographic findings.

Foreign bodies in the extremities are a common complaint in agrarian populations. Most of these patients have normal X-rays as these foreign bodies being radiolucent. Ultrasonography being readily available and cheap modality could be a very useful tool to confirm the presence of foreign body, determine its depth, size and proximity to adjoining nerve, vessel, or a tendon. This will minimize the false-negative surgical explorations and prevent damage to adjoining structures. Although pre-op CT scan or MRI could be the better option but considering the availability and financial constrains especially in developing and underdeveloped nations, ultrasonography seems to be a useful tool to aid in exploration. Because of lack of comparison, we cannot recommend USG over CT scan or MRI, and this could be the possible limitation of our study.
